# Synthesis of quinoline/naphthalene-containing azaphenothiazines and their potent in vitro antioxidant properties

**DOI:** 10.1007/s00044-014-1247-y

**Published:** 2014-09-12

**Authors:** Małgorzata Jeleń, Eugenia I. Bavavea, Maria Pappa, Angeliki P. Kourounakis, Beata Morak-Młodawska, Krystian Pluta

**Affiliations:** 1 Department of Organic Chemistry, School of Pharmacy with the Division of Laboratory Medicine, The Medical University of Silesia, Jagiellońska 4, 41-200 Sosnowiec, Poland; 2 Department of Medicinal Chemistry, Faculty of Pharmacy, University of Athens, 15771 Athens, Greece

**Keywords:** Phenothiazine derivatives, NH-azaphenothiazines, Quinonaphthothiazines, Diquinothiazines, Lipid peroxidation, Lipophilicity

## Abstract

New tetracyclic and pentacyclic azaphenothiazines containing one or two quinoline rings instead of benzene rings were obtained in the original reactions of isomeric diquinodithiins, dichlorodiquinolinyl sulfides, and disulfide with aromatic amines. The type of ring fusion in the azaphenothiazine system was concluded from the ^1^H NMR spectra. The obtained azaphenothiazines were evaluated in vitro for their antioxidant activity on rat hepatic microsomal membranes for protection of non-enzymatic lipid peroxidation promoted by the Fe^2+^/ascorbic acid redox system. Most compounds exhibited a very significant antioxidant activity with IC_50_ values between 1 and 23 μM. The degree of antioxidant activity depends on the lipophilicity and molecular size as well as the (non)substitution of the thiazine nitrogen atom and type of ring system fusion. It is the first time to our knowledge that azaphenothiazines are shown to exhibit such potent antioxidant activity.

## Introduction

Phenothiazines are an important class of drugs exhibiting antipsychotic, antihistaminic, antitussive, and anti-emetic activities (Gupta and Kumar, 1988). The most significant modifications of the phenothiazine structure are the introduction of new pharmacophoric substituents at the thiazine nitrogen atom and the substitution of the benzene rings with other homoaromatic or heteroaromatic ones. Recently studied phenothiazines exhibit promising antibacterial, antifungal, anticancer, antiviral, anti-inflammatory, antimalarial, antifilarial, trypanocidal, anticonvulsant, analgesic, immunosuppressive, and multidrug resistance reversal properties (Aaron *et al*., 2009; Dasgupta *et al*., 2008; Motohashi *et al*., 2006; Pluta *et al*., 2011). In our study of new azaphenothiazines, we elaborated the synthesis of new types of phenothiazines containing the heterocyclic rings of pyridine or quinoline. Some of those azaphenothiazines exhibited promising immunosuppressive and anticancer activities against cell lines of ten types of human cancer in vitro: leukemia, non-small cell lung cancer, melanoma, as well as colon, CNS, ovarian, renal, prostate, breast, and skin cancer (Jeleń *et al*., 2013; Pluta *et al*., 2010; Zimecki *et al*., 2009).

Free radicals, generated in many redox processes, may induce oxidative damage of proteins, lipids, and DNA. They affect living cells and mediate the pathogenesis of many chronic diseases, such as atherosclerosis, Parkinson’s and Alzheimer’s diseases, stroke, and arthritis, acting by various mechanisms. A recent trend in the field of antioxidant development focuses on multipotent antioxidant agents that not only can prevent biological substrates from radical induced oxidative damage but also possess additional pharmacological properties (Zhang *et al*., 2006). The study of antioxidant activity among N-heterocycles has attracted attention. One such heterocyclic structural scaffold is the 1,4-thiazine ring present in the multi-target phenothiazines. Therefore, recent reports on promising antioxidant compounds deal with classical and new phenothiazines (Asghar *et al*., 2012; Borges *et al*., 2010; Liu *et al*., 2009; Naik *et al*., 2012;) and their derivatives, benzothiazines (Matralis *et al*., 2011), and azaphenothiazines (Kumar *et al*., 2010; Morak-Młodawska *et al*., 2010).

Our previous work (Morak-Młodawska *et al*., 2010) revealed that tricyclic azaphenothiazines being dipyridothiazines have a variable degree of antioxidant activity depending on substitution at the thiazine nitrogen atom, with the unsubstituted compound being the most active. In this study, we obtained eleven tetracyclic and pentacyclic (linearly and angularly fused) azaphenothiazines containing one or two quinoline rings instead of the benzene rings and determined their antioxidant properties to find an influence of the number of rings, their type of fusion, and their substituents.

## Materials and methods

### General techniques

Melting points were determined in open capillary tubes on a Boetius melting point apparatus and were uncorrected. The ^1^H NMR spectra were recorded on a Bruker Fourier 300 and a Bruker DRX spectrometer at 500 MHz in CDCl_3_ and DMSO-*d*_6_ with tetramethylsilane as the internal standard. The ^13^C NMR spectra were recorded at 75 MHz. Electron impact (EI MS) mass spectra were run on a Finnigan MAT 95 spectrometer at 70 eV. The thin-layer chromatography was performed on aluminum oxide 60 F_254_ neutral (type E, Merck 1.05581) with CH_2_Cl_2_ and on silica gel 60 F_254_ (Merck 1.05735) with CHCl_3_-EtOH (10:1 v/v) as eluents.

### Synthesis of substrates **1**, **2**, **7**, **8**, **10**, and **11**

The substrates for the title compounds, i.e., diquinodithiins **1**, **7**, **10**, sulfides **8**, **11**, and disulfide **2**, were obtained as described previously (Nowak *et al*., 2002, 2003, 2007; Pluta, 1994).

### Quino[3,2-b]benzo[1,4]thiazines (**3a**–**c**)

#### From diquino-1,4-dithiin **1**

A mixture of diquino-1,4-dithiin **1** (0.16 g, 0.5 mmol) and hydrochloride of aniline, or *p*-chloroaniline or *p*-methoxyaniline (2.5 mmol) was finely powdered together and then heated on an oil bath at 200–205 °C for 4 h and after cooling water was added (10 ml) and the insoluble solid was filtered off. The filtrate was alkalized with 5 % aqueous sodium hydroxide to pH 10, and the resulting solid was filtered off and washed with water. The combined solids were purified by column chromatography (silica gel, CHCl_3_) to give quinobenzothiazines **3a**–**c**.

##### 6*H*-Quinobenzothiazine (**3a**)

0.06 g (24 %), yellow, mp 169–170 °C (mp 169–170 °C, Jeleń and Pluta, 2009). ^1^H NMR (CDCl_3_) *δ*: 6.62 (m, 1H, H-7), 6.87 (m, 1H, H-9), 7.03 (m, 2H, H-8, H-10), 7.26 (t, 1H, H-2), 7.47 (m, 2H, H-1, H-3), 7.53 (s, 1H, H-12), 7.56 (d, 1H, H-4). ^13^C NMR (CDCl_3_) *δ*: 115.57 (C-7), 116.49 and 116.69 (C-10a, C-11a), 122.95 (C-9), 124.19 (C-2), 125.86 (C-10), 126.04 and 126.45 (C-1, C-8), 126.56 (C-12a), 127.57 (C-4), 129.52 (C-3), 131.69 (C-12), 138.45 (C-6a), 145.40 (C-4a), 150.98 (C-5a).

##### 6H-9-Chloroquinobenzothiazine (**3b**)

0.08 g (28 %), yellow, mp 224–225 °C (mp 224–225 °C, Jeleń and Pluta, 2009). ^1^H NMR (CDCl_3_) *δ*: 6.63 (d, 1H, H-7), 6.99 (s, 1H, H-10), 7.01 (d, 1H, H-8), 7.33 (t, 1H, H-2), 7.51 (d, 1H, H-1), 7.52 (t, 1H, H-3), 7.59 (d, 1H, H-4), 7.60 (s, 1H, H-12). ^13^C NMR (CDCl_3_) *δ*: 115.80 (C-11a), 116.71 (C-7), 118.19 (C-10a), 124.84 and 124.91 (C-8, C-10), 125.65 (C-2), 126.13 (C-12a), 126.61 (C-1), 127.59 (C-4), 128.56 (C-9), 130.31 (C-3), 132.35 (C-12), 136.29 (C-6a), 143.81 (C-4a), 150.04 (C-5a),

##### 6*H*-9-Methoxyquinobenzothiazine (**3c**)

0.09 g (32 %), orange, mp 159–160 °C.

^1^H NMR (CDCl_3_) *δ* 3.76 (s, 3H, CH_3_), 6.54 (d, 1H, H-7), 6.63 (d, 1H, H-10), 6.76 (d, 1H, H-8), 7.29 (t, 1H, H-2), 7.46 (d, 1H, H-1), 7.52 (t, 1H, H-3), 7.55 (s, 1H, H-12), 7.57 (d, 1H, H-4). ^13^C NMR (CDCl_3_) *δ*: 111.59 (C-10), 113.22 (C-8), 116.41 (C-11a), 116.82 (C-7), 117.39 (C-10a), 124.36 and 124.49 (C-1, C-2), 125.80 (C-12a), 126.55 (C-4), 130.10 (C-3), 130.60 (C-6a), 132.07 (C-12), 143.40 (C-4a), 150.36 (C-5a), 156.12 (C-9). EIMS *m*/*z*: 280 (M^+^, 100), 265 (M-CH_3_, 90). Anal. Calcd. for C_16_H_12_N_2_OS: C, 68.55; H, 4.31; N, 9.99. Found: C, 68.45; H, 4.36; N, 9.82.

#### From 2,2′-dichloro-3,3′-diquinolinyl disulfide (**2**)

A solution of disulfide **2** (0.20 g, 0.5 mmol) and *p*-methoxyaniline (0.25 g, 2 mmol) in monomethyl ether of diethylene glycol (MEDG) (5 ml) was refluxed for 3 h. After cooling, the solution was poured into water (20 ml) and alkalized with 5 % aqueous sodium hydroxide to pH 10. The resulting solid was filtered off, washed with water, and purified by column chromatography (silica gel, CHCl_3_) to give 0.18 g (64 %) of 6*H*-9-methoxyquinobenzothiazine (**3c**).

### Quino[3,2-b]naphtho[1′,2′-e][1,4]thiazine (**4**)

Diquinodithiin **1** (0.16 g, 0.5 mmol) was finely powdered together with 1-naphthylamine hydrochloride (0.45 g, 2.5 mmol) on an oil bath at 200–205 °C for 4 h. After cooling, the solution was poured into water (10 ml) and alkalized with 5 % aqueous sodium hydroxide to pH 10. The resulting solid was filtered off, washed with water, and purified by column chromatography (Al_2_O_3_, CHCl_3_) to give 0.08 g (27 %) of 14*H*-quinonaphthothiazine (**4**), orange, mp 147-148 °C.

^1^H NMR (CDCl_3_) *δ*: 7.01 (d,1H, H-6), 7.30 (t, 1H, H-10), 7.47 (m, 4H, H-3, H-4, H-5, H-9), 7.52 (t, 1H, H-2), 7.56 (s, 1H, H-8), 7.60 (t, 1H, H-11), 7.64 (d, 1H, H-12), 7.75 (d, 1H, H-1). ^13^C NMR (CDCl_3_) *δ*: 110.98 (C-6a), 116.91 (C-7a), 118.43 (C-1), 121.89 (C-14b), 122.87 (C-6), 123.70 (C-5), 124.49 (C-10), 125.93, 126.45 and 126.83 (C-2, C-3, C-9), 126.90 (C-8a), 128.92 and 129.65 (C-4, C-12), 131.54 (C-11), 132.55 (C-4a), 133.04 (C-8), 135.07 (C-14a), 145.23 (C-12a), 150.98 (C-13a). EIMS *m*/*z*: 300 (M^+^, 100), 268 (M-S, 45). Anal. Calcd. for C_19_H_12_N_2_S: C, 75.97; H, 4.03; N, 9.33. Found: C, 75.82; H, 4.07; N, 9.21.

### Quino[3,2-b]naphtho[2′,1′-e][1,4]thiazine (**5**)

Diquinodithiin **1** (0.16 g, 0.5 mmol) was finely powdered together with 2-naphthylamine hydrochloride (0.45 g, 2.5 mmol) on an oil bath at 200–205 °C for 4 h. After cooling, the solution was poured into water (10 ml) and alkalized with 5 % aqueous sodium hydroxide to pH 10. The resulting solid was filtered off, washed with water, and purified by column chromatography (Al_2_O_3_, CHCl_3_) to give 0.12 g (40 %) of 7*H*-quinonaphthothiazine (**5**), greenish, mp 244-245 °C.

^1^H NMR (CDCl_3_) *δ*: 7.06 (d, 1H, H-6), 7.37 (t, 1H, H-11), 7.47 (t, 1H, H-3), 7,57 (m, 3H, H-2, H-10, H-12), 7.65 (d, 1H, H-5), 7.66 (d, 1H, H-4), 7.72 (s, 1H, H-13), 7.80 (m, 2H, H-9, H-1). ^13^C NMR (CDCl_3_) *δ*: 107.94 (C-14a), 115.77 (C-13a), 116.04 (C-6), 121.32 (C-1), 123.33, 123.66 and 123.89 (C-3, C-9, C-11), 125.23 (C-12a), 125.62 (C-2), 126.36, 126.99 and 127.56 (C-4, C-5, C-12), 128.73 (C-4a), 129.22 (C-10), 129.62 (C-14b), 131.51 (C-13), 133.54 (C-6a), 142.13 (C-8a), 149.64 (C-7a). EIMS *m*/*z*: 300 (M^+^, 100), 268 (M-S, 50). Anal. Calcd. for C_19_H_12_N_2_S: C, 75.97; H, 4.03; N, 9.33. Found: C, 75.88; H, 4.05; N, 9.19.

### Diquino[3,2-b;6′,5′-e][1,4]thiazine (**6**)

Diquinodithiin **1** (0.16 g, 0.5 mmol) was finely powdered together with 6-aminoquinoline hydrochloride (0.46 g, 2.5 mmol) on an oil bath at 200–205 °C for 4 h. After cooling, the solution was poured into water (10 ml) and alkalized with 5 % aqueous sodium hydroxide to pH 10. The resulting solid was filtered off, washed with water, and purified by column chromatography (Al_2_O_3_, CHCl_3_) to give 0.10 g (33 %) of 7*H*-diquinothiazine (**6**), brown, mp 260–261 °C.

^1^H NMR (CDCl_3_) *δ*: 7.44 (t, 1H, H-11), 7.49 (d, 1H, H-6), 7.57 (m, 2H, H-2, H-12), 7.64 (t, 1H, H-10), 7.70 (d, 1H, H-9), 7.75 (s, 1H, H-13), 8.10 (d, 1H, H-5), 8.19 (d, 1H, H-1), 8.90 (d, 1H, H-3). ^13^C NMR (CDCl_3_) *δ*: 107.62 (C-14a), 114.59 (C-13a), 119.33 (C-6), 120.76 (C-2), 124.05 (C-11), 124.37 and 125.45 (C-12a, C-14b), 125.65 (C-12), 128.27, 129.24, 129.62 and 129.64 (C-1, C-5, C-9, C-10), 131.80 (C-13), 134.54 (C-6a), 144.53 (C-7a), 147.55 (C-3), 149.49 and 149.55 (C-4a, C-8a). EIMS *m*/*z*: 301 (M^+^, 100), 269 (M-S, 45). Anal. Calcd. for C_18_H_11_N_3_S: C, 71.74; H, 3.68; N, 13.94. Found: C, 71.59; H, 3.71; N, 13.72.

### Diquino[3,2-b;2′,3′-e][1,4]thiazines (**9**)

#### 6*H*-Diquinothiazine **9a**

This compound was obtained in the reaction of diquinodithiin **7** with acetamide (Nowak *et al*., 2007), orange, mp > 300 °C (mp > 300 °C, Nowak *et al*., 2007). ^1^H NMR (CDCl_3_) *δ*: 7.42 (t, 2H, H-2, H-10), 7.55 (d, 2H, H-1, H-11), 7.62 (t, 2H, H-3, H-9), 7.72 (s, 2H, H-12, H-14), 7.86 (d, 2H, H-4, H-8). ^13^C NMR (DMSO-*d*_6_) *δ*: 124.83 (C-12a, C-13a), 127.29 (C-2, C-10), 128.00 (C-11a, C-14a), 128.16 and 128.28 (C-1, C-11 and C-4, C-8), 131.29 (C-3, C-9), 135.26 (C-12, C-14), 146.58 (C-4a, C-7a), 156.22 (C-5a, C-6a).

#### 6-(*p*-Fluorophenyl)diquinothiazine (**9b**)

##### From diquinodithiin **7**

Diquinodithiin **7** (0.16 g, 0.5 mmol) was finely powdered together with *p*-fluoroaniline hydrochloride (0.37 g, 2.5 mmol), and the mixture was heated on an oil bath at 200–205 °C for 3 h. After cooling, water (10 ml) was added to the reaction mixture and the resulting solid was filtered off, washed with water, air-dried, and purified by column chromatography (Al_2_O_3_, CH_2_Cl_2_) to give 0.14 g (35 %) of 6-(*p*-fluorophenyl)diquinothiazine (**9b**), yellow, mp 248–249 °C.

##### From 2,2′-dichloro-3,3′-diquinolinyl sulfide **8**

A solution of sulfide **8** (0.18 g, 0.5 mmol) and *p*-fluoroaniline (0.17 g, 1.5 mmol) in MEDG (5 ml) was refluxed for 3 h. After cooling, the solution was poured into water (20 ml) and alkalized with 5 % aqueous sodium hydroxide to pH = 10. The resulting solid was filtered off, washed with water, and purified by column chromatography (Al_2_O_3_, CH_2_Cl_2_) to give 0.16 g (81 %) 6-(*p*-fluorophenyldiquinothiazine (**9b**), yellow, mp 248–249 °C.

^1^H NMR (CDCl_3_) *δ*: 7.31 (m, 4H, H-2, H-10, C_6_H_2_), 7.47 (m, 4H, H-3, H-9, C_6_H_2_), 7.56 (d, 2H, H-1, H-11), 7.67 (d, 2H, H-4, H-8), 7.83 (s, 2H, H-12, H-14). ^13^C NMR (CDCl_3_) *δ*: 115.85 (*J* = 22.6 Hz, *m*-C of C_6_H_4_F), 115.98 (C-12a, C-13a), 125.16 (C-2, C-10), 125.78 (C-11a, C-14a), 125.96 (C-1, C-11), 128.07 (C-4, C-8), 129.37 (C-3, C-9), 132.07 (C-12, C-14), 132.40 (*J* = 7.5 Hz, *o*-C of C_6_H_4_F), 135.59 (*J* = 2.5 Hz, *ipso*-C of C_6_H_4_F), 145.13 (C-4a, C-7a), 150.98 (C-5a, C-6a), 161.83 (*J* = 244.6 Hz, *p*–C of C_6_H_4_F).

EIMS *m*/*z*: 395 (M^+^, 75), 394 (M-1, 100), 363 (M-S, 5). Anal. Calcd. for C_24_H_14_FN_3_S: C, 72.89; H, 3.57; N, 10.63. Found: C, 72.80; H, 3.55; N, 10.41.

### Diquino[3,4-b;4′,3′-e][1,4]thiazines (**12a**–**c**)

6*H*-Diquinothiazine (**12a**) and 6-methyldiquinothiazine (**12b**) were obtained from the reaction of sulfide **11** with ammonia and methylamine in hot phenol (Pluta, 1997).

#### 6*H*-Diquinothiazine (**12a**)

Beige, mp 200–201 °C (mp 200–201 °C, Pluta, 1997). ^1^H NMR (CDCl_3_) *δ*: 7.64 (t, 2H, H-2, H-12), 7.71 (t, 2H, H-3, H-11), 7.81 (d, 2H, H-4, H-10), 8.04 (d, 2H, H-1, H-13), 8.40 (s, 2H, H-6, H-8). ^13^C NMR (CDCl_3_) *δ*: 109.10 (C-6a, C-7a), 117.18 (C-13a, C-14b), 117.41 (C-1, C-13), 127.25 (C-2, C-12), 129.49 (C-3, C-11), 130.78 (C-4, C-10), 142.21 (C-4a, C-9a), 147.94 (C-6, C-8), 148.07 (C-13b, C-14a).

#### 6-Methyldiquinothiazine (**12b**)

Yellow, mp 156–157 °C (mp 156–157 °C, Pluta, 1997). ^1^H NMR (CDCl_3_) *δ*: 3.54 (s, 3H, CH_3_), 7.66 (t, 2H, H-2, H-12), 7.72 (t, 2H, H-3, H-11), 8.11 (d, 2H, H-4, H-10), 8.34 (d, 2H, H-1, H-13), 8.66 (s, 2H, H-6, H-8). ^13^C NMR (CDCl_3_) *δ*: 43.63 (CH_3_), 122.09 (C-1, C-13), 124.17 and 124.42 (C-6a, C-7a and C-13a, C-14b), 127.46 (C-2, C-12), 129.44 (C-3, C-11), 130.11 (C-4, C-10), 148.33 (C-6, C-8), 148.76 and 148.85 (C-4a, C-9a and C-13b, C-14a).

### 14-(*p*-Fluorophenyl)diquinothiazine (**12c**)

#### From diquinodithiin **10**

Diquinodithiin **10** (0.16 g, 0.5 mmol) was finely powdered together with *p*-fluoroaniline hydrochloride (0.37 g, 2.5 mmol), and the mixture was heated on an oil bath at 200–205 °C for 3 h. After cooling, water (10 ml) was added to the reaction mixture and the resulting solid was filtered off, washed with water, air-dried, and purified by column chromatography (Al_2_O_3_, CH_2_Cl_2_) to give 0.12 g (30 %) of 14-(*p*-fluorophenyl)diquinothiazine (**12c**), beige, mp 315-316 °C.

#### From 4,4′-dichloro-3,3′-diquinolinyl sulfide (**11**)

A solution of sulfide **11** (0.18 g, 0.5 mmol) and *p*-fluoroaniline (0.17 g, 1.5 mmol) in MEDG (5 mL) was refluxed for 3 h. After cooling, the solution was poured into water (20 ml) and alkalized with 5 % aqueous sodium hydroxide to pH 10. The resulting solid was filtered off, washed with water and purified by column chromatography (Al_2_O_3_, CHCl_3_) to give 0.17 g (86 %) of 14-(*p*-fluorophenyl)diquinothiazine (**12c**), beige, mp 315–316 °C.

^1^H NMR (CDCl_3_) *δ*: 6.43 (dd, 2H, C_6_H_2_), 6.77 (m, 2H, C_6_H_2_), 7.75 (t, 2H, H-2, H-12), 7.85 (t, 2H, H-3, H-11), 8.34 (d, 2H, H-4, H-10), 8.39 (d, 2H, H-1, H-13), 9,06 (s, 2H, H-6, H-8). ^13^C NMR (CDCl_3_) *δ*: 115.75 (*J* = 22.5 Hz, *m*-C of C_6_H_4_F), 116.30 (*J* = 7.5 Hz, *o*-C of C_6_H_4_F), 122.87 (C-1, C-13), 126.82 (C-13a, C-14b), 128.51 (C-2, C-12), 129.89 (C-6a, C-7a), 130.13 (C-3, C-11), 130.25 (C-4, C-10), 140.57 (*J* = 2.5 Hz, *ipso*-C of C_6_H_4_F), 145.54 (C-13b, C-14a), 147.98 (C-4a, C-9a), 149.49 (C-6, C-8), 158.07 (*J* = 238.5 Hz, *p*–C of C_6_H_4_F). EIMS *m*/*z*: 395 (M^+^, 100), 363 (M-S,20), 300 (M-C_6_H_4_F, 17). Anal. Calcd. for C_24_H_14_FN_3_S: C, 72.89; H, 3.57; N, 10.63. Found: C, 72.77; H, 3.59; N, 10.46.

### In vitro lipid peroxidation

Heat-inactivated hepatic microsomes from untreated rats were prepared as described (Rekka *et al*., 1989). The incubation mixture contained microsomal fraction (corresponding to 2.5 mg of hepatic protein per ml or 4 mM fatty acid residues), ascorbic acid (0.2 mM) in Tris–HCl/KCl buffer (50 mM/150 mM, pH 7.4), and the studied compounds (50–1 μM) dissolved in DMSO. The reaction was initiated by addition of a freshly prepared FeSO_4_ solution (10 μΜ), and the mixture was incubated at 37 °C for 45 min. Lipid peroxidation of aliquots was assessed spectrophotometrically (535 against 600 nm) as TBAR. Both compounds and solvents were found not to interfere with the assay. Each assay was performed in duplicate, and IC_50_ values represent the mean concentration of compounds that inhibit the peroxidation of control microsomes by 50 % after 45 min of incubation. All standard errors are within 10 % of the respective reported values.

### Calculation of lipophilicity, molecular mass, surface area, and molecular volume

Lipophilicity (as cLogP), molecular mass (M), surface area (S), and molecular volume (V_M_) were calculated using CS Chem 3D Ultra 7.0 (CambridgeSoft) and Spartan’04 (Wavefunction, Inc. Irvine, CA).

## Results and discussion

### Synthesis

**Scheme 1:**
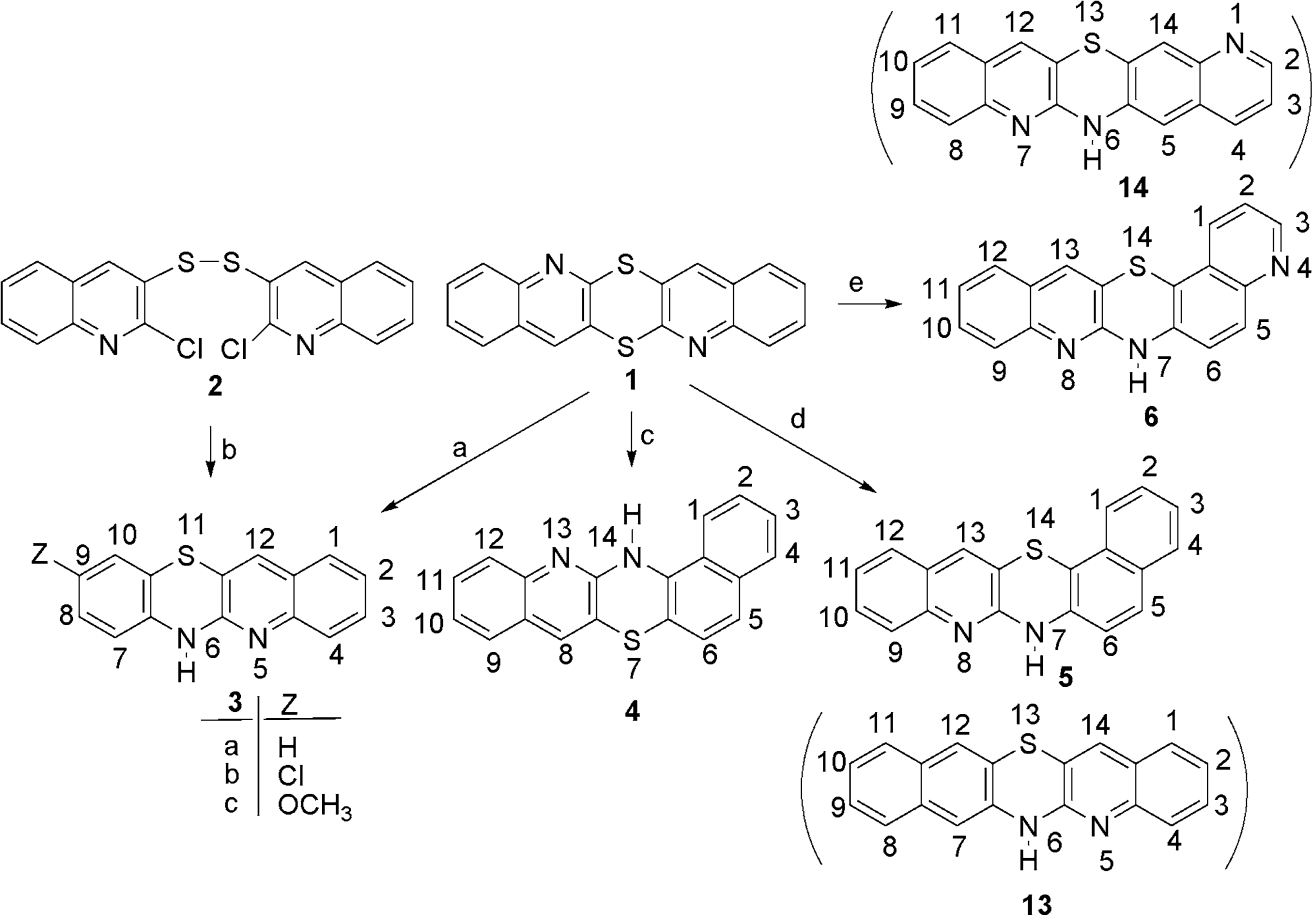
Reactans: **a** C_6_H_5_NH_2_·HCl (*p*-ClC_6_H_4_NH_2_·HCl, *p*-CH_3_OC_6_H_4_NH_2_·HCl), 200–205 °C, 4 h; **b** *p*-CH_3_OC_6_H_4_NH_2_, MEDG, reflux, 3 h; **c** 1-naphthylamine**·**HCl, 200–205 °C, 4 h; **d** 2-naphthylamine·HCl, 200–205 °C, 4 h; **e** 6-aminoquinoline·HCl, 200–205 °C, 4 h

**Scheme 2:**
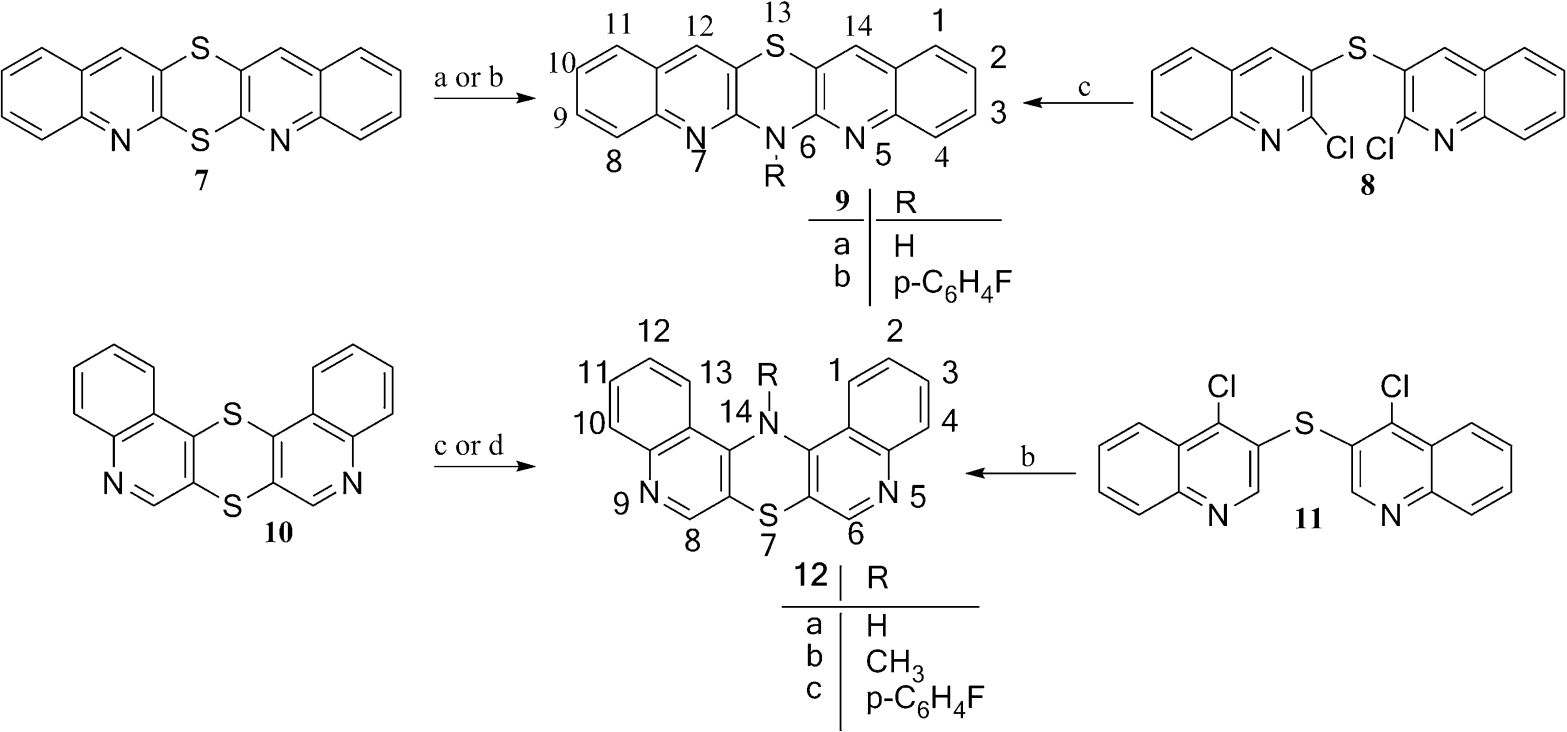
Reactans: **a** CH_3_CONH_2_, K_2_CO_3_, 180 °C, 0.5 h; **b** *p*F-C_6_H_4_NH_2_·HCl), 200–205 °C, 3 h; **c** *p*-FC_6_H_4_NH_2_, MEDG, reflux, 3 h; **d** NH_3_ (CH_3_NH_2_), phenol, 180 °C, 1 h

The synthesis of the title azaphenothiazines was based on the reactions of isomeric diquinodithiins, dichlorodiquinolinyl sulfides, and disulfide with amines, ammonia, and acetamide. The fusion reactions of linearly condensed diquinodithiin **1** with hydrochlorides of aniline and its *p*-substituted derivatives such as *p*-chloroaniline and *p*-methoxyaniline led to tetracyclic 9-substituted 6*H*-quinobenzothiazines **3a**–**c** (Scheme 1). 9-Methoxy-6*H*-quinobenzothiazine **3c** was obtained in better yield in the reaction of 2,2′-dichloro-3,3′-diquinolinyl disulfide **2** with *p*-methoxyaniline in monomethyl ether of diethylene glycol. The similar reaction of diquinodithiin **1** with hydrochlorides of 1-naphthylamine, 2-naphthylamine, and 6-aminoquinoline gave pentacyclic 7*H*-quinonaphthothiazine **4**, 14*H*-quinonaphthothiazine **5**, and 7*H*-diquinothiazine **6**. The reaction of isomeric diquinodithiin **7** with acetamide and *p*-fluoroaniline hydrochloride gave linearly condensed pentacyclic 6*H*-diquinothiazines **9a** and 6-(*p*-fluorophenyl)diquinothiazine **9b** (Scheme 2). Analogous reaction of another isomeric diquinodithiin **10** with *p*-fluoroaniline hydrochloride led to angularly condensed diquinothiazine **12c**. Better yields of the fluoroaniline products **9b** and **12c** were achieved when x,x’-dichloro-3,3′-diquinolinyl sulfides **8** and **11** (*x* = 2 and 4) were used. Sulfide **11** reacted also with ammonia or methylamine in hot phenol to give diquinothiazines **12a**, **b**.

The described syntheses were monitored by TLC analysis. All chromatograms of new compounds showed characteristic for azaphenothiazines (Jeleń *et al*., 2011) color changing during irradiation with UV light from blue to yellow (**4**, **9b**), from yellow to green (**5**, **6**), from orange to yellow (**12c**), and from yellow to orange (**7c**).

### Structure

It is well known that the synthesis of phenothiazines can proceed via the Smiles rearrangement of the S–N type of the appropriate sulfide (Pluta *et al*., 2009). The identification of the product structures was based on the spectroscopic ^1^H NMR and MS analysis. In the case of the reactions of sulfides **7** and **11**, the products **9** and **12** possessed the C_2v_ symmetry (the left part was a mirror image of the right one) what excluded the stage of rearrangement. The reactions of diquinodithiin **1** and disulfide **2** with anilines proceeded similarly without the stage of rearrangement to give tetracyclic quinobenzothiazines **3a**–**c** (Jeleń and Pluta, 2009). The reaction with 1-naphthylamine gave pentacyclic quinonaphthothiazine **4**. On the contrary, the reactions with 2-naphthylamine and 6-aminoquinoline were more complex as there were two possibilities of the thiazine ring formation. The ^1^H NMR analysis of the reaction products pointed at compounds **5** and **6** excluding compounds **13** and **14**, as evidenced from coupling constants; the H-5 and H-6 protons in compounds **5** and **6** showed a coupling constant *J*_ortho_, whereas analogous protons in compounds **13** and **14** (H-7/H-12 and H-5/H-14, respectively) would have shown a coupling constant *J*_para_, which is very small (i.e., *J*_1,4_ = 0.6-0.8 Hz in naphthalene (Hamm and von Philipsborn, 1971; Lucchini and Wells, 1976) and *J*_5,8_ = 0.5-0.8 Hz in quinoline (Hamm and von Philipsborn, 1971; Jones, 1977). We did not observe such small values of coupling constants in the reaction products **5** and **6**.

### Antioxidant activity

**Fig. 1:**
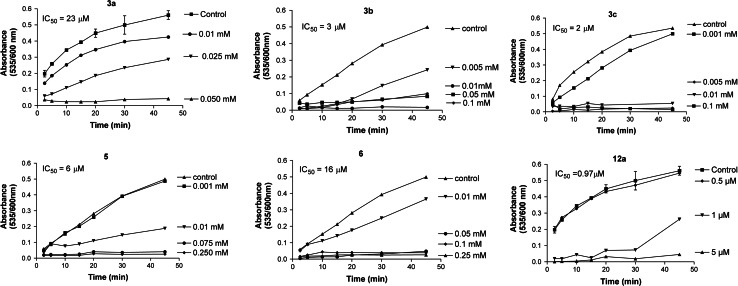
Representative *graphs* of the time course of lipid peroxidation as affected by various concentrations of compounds **3a**–**c**, **5**, **6**, and **12a**. IC_50_ values are calculated according to these results as the concentration showing 50 % inhibition of the lipid peroxidation reaction at 45 min incubation time

The effect of the new derivatives on non-enzymatic lipid peroxidation of rat hepatic microsomal membrane lipids was investigated in vitro. Most of the studied derivatives demonstrated significant antioxidant activity, with IC_50_ values between 1 and 23 μM (Table 1). It is worthwhile to mention that under the same experimental conditions known potent antioxidants, trolox ((S)-(-)-6-hydroxy-2,5,7,8-tetramethylchromane-2-carboxylic acid) and probucol (4,4′-[(1-methylethylidene)bis(thio)]bis[2,6-bis(1,1-dimethylethyl)phenol]), exhibited IC_50_ values of 25 μM and >1 mM, respectively (Kourounakis *et al*., 2008). Further, all of the active new derivatives were significantly much more potent than previously studied tricyclic dipyridothiazines (IC_50_ of most active compounds was between 64 and 470 μM) (Morak-Młodawska *et al*., 2010). The time course of lipid peroxidation, as affected by various concentrations of representative compounds, is depicted in Fig. 1.

**Table 1:** IC_50_ values for in vitro lipid peroxidation (LP), LogP, molecular volume (V_M_), and molecular mass (M) as well as surface area (S) of the tested compounds

Compound	LP IC_50_ (μM)	LogP	M	S (Å^2^)	V_M_ (Å^3^)
**3a**	23	3.37	250.06	253.13	246.02
**3b**	3	3.93	284.02	268.84	259.50
**3c**	2	3.25	280.07	283	273.38
**4**	2	4.37	300.07	297.74	296.96
**5**	6	4.37	300.07	297.68	296.87
**6**	16	3.46	301.07	293.28	291.10
**9a**	>1000	4.20	301.07	295.91	291.54
**9b**	>1000	6.00	395.09	374.91	379.66
**12a**	1	2.71	301.07	291.11	290.87
**12b**	500	4.77	315.08	317.08	321.82
**12c**	>1000	4.51	395.09	359.77	375.69

Tetracyclic NH-azaphenothiazines **3a**–**c** exhibited significant activity dependent on the substitution (H, Cl, and OCH_3_) on the benzene ring (Table 1). From the pentacyclic compounds, the angularly fused with unsubstituted, the thiazine nitrogen atom (**4**–**6** and **12a**) exhibited very significant activity with most active compound **12a**, which showed an IC_50_ of 1 μM. The change of the quinoline moiety into naphthalene (compare compounds **4** and **5** with **6**) marginally increased activity. However, compounds with a linearly fused ring system (**9a** and **9b**) and/or a large aryl substituent at the thiazine nitrogen atom (**9b** and **12c**) did not show any antioxidant activity, while compound **12b**, with a small substituent, exhibited very weak activity.

Considering three isomers (**6**, **9a**, and **12a**), one can find that their antioxidant activity increased with decreasing lipophilic character represented by the logP values. On the other hand, the least active compounds (**9b**, **12b**, and **12c**) exhibited high values of molecular descriptors such as molecular mass (M > 315), molecular volume (*V*_M_ > 321), and surface area (S > 317, Table 1). However, attempts to the correlate the activity with those properties turned out to be unsatisfactory.

In conclusion, eleven tetracyclic and pentacyclic (linearly or angularly condensed) azaphenothiazines were synthesized, and structure–(antioxidant)activity relationships were investigated. The type of the ring fusion was concluded from the ^1^H NMR spectra. The degree of antioxidant activity of these derivatives seems to depend on their lipophilicity and molecular mass. The non-substitution of the thiazine nitrogen atom, the type of ring system fusion, and the nature of substituents promote activity. Finally, it is the first time to our knowledge that azaphenothiazines are shown to exhibit such potent antioxidant activity.
